# Unresolved issues in pre-meiotic anther development

**DOI:** 10.3389/fpls.2014.00347

**Published:** 2014-07-21

**Authors:** Timothy Kelliher, Rachel L. Egger, Han Zhang, Virginia Walbot

**Affiliations:** ^1^Syngenta Biotechnology Inc., Research Triangle ParkNC, USA; ^2^Department of Biology, Stanford UniversityStanford, CA, USA

**Keywords:** arabidopsis, rice, maize, cell fate specification, tapetum, meiosis, phased small RNA

## Abstract

Compared to the diversity of other floral organs, the steps in anther ontogeny, final cell types, and overall organ shape are remarkably conserved among Angiosperms. Defects in pre-meiotic anthers that alter cellular composition or function typically result in male-sterility. Given the ease of identifying male-sterile mutants, dozens of genes with key roles in early anther development have been identified and cloned in model species, ordered by time of action and spatiotemporal expression, and used to propose explanatory models for critical steps in cell fate specification. Despite rapid progress, fundamental issues in anther development remain unresolved, and it is unclear if insights from one species can be applied to others. Here we construct a comparison of Arabidopsis, rice, and maize immature anthers to pinpoint distinctions in developmental pace. We analyze the mechanisms by which archesporial (pre-meiotic) cells are specified distinct from the soma, discuss what constitutes meiotic preparation, and review what is known about the secondary parietal layer and its terminal periclinal division that generates the tapetal and middle layers. Finally, roles for small RNAs are examined, focusing on the grass-specific phasiRNAs.

## Introduction

**Figure 1:**
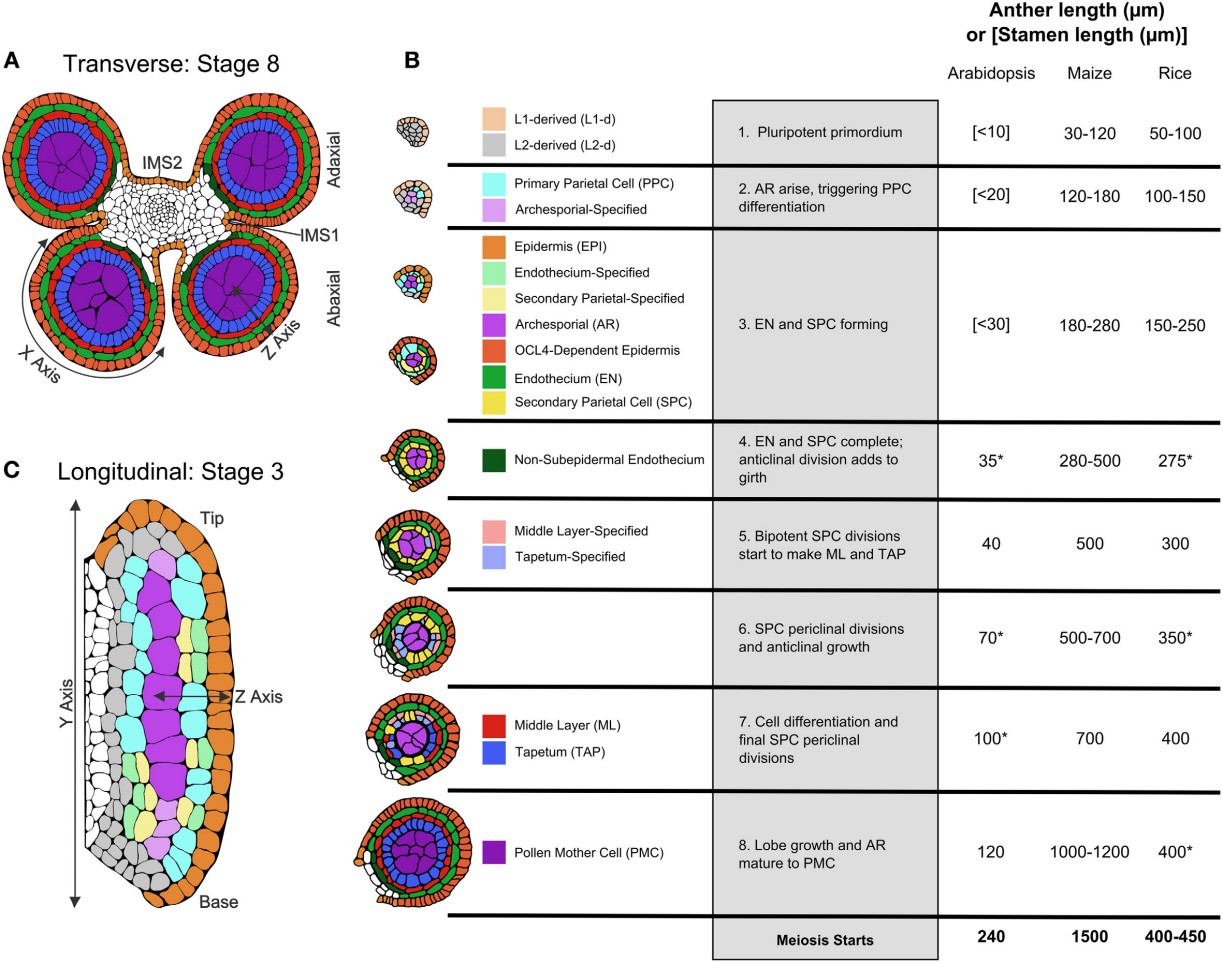
**Pre-meiotic anther development. (A)** The four-lobed anther typical of flowering plants with a central column of vasculature that extends into the stamen filament surrounded by connective tissue [stage 8]. **(B)** Tracings of confocal images of single lobes of the W23 maize inbred are colorized to show the progression of cell fate specification and anther lobe patterning. At stage [1] the lobe consists of pluripotent Layer1- and Layer2-derived cells, colored in beige and light gray, respectively. For all cell types, just-specified cells are colorized in a pale shade, which gradually darkens as the cells acquire stereotyped differentiated shapes, volumes, and staining properties. The first specification event results in visible archesporial (AR) cells centrally within each lobe. In maize, the glutaredoxin encoded by *Msca1* responds to growth-generated hypoxia to initiate AR differentiation, marked by secretion of the MAC1 protein, which is required for cell specification of the subepidermal L2-d cells to Primary Parietal Cells (PPC) [stage 2]. PPC divide periclinally generating the subepidermal Endothecium (EN) and the bipotent Secondary Parietal Cells (SPC). In the same timeframe, Epidermal (EPI) cells differentiate; signals controlled by expression of the OCL4 epidermal-specific transcription factor suppress excess periclinal divisions in the EN (Vernoud et al., 2009) [stage 3]. Following these early patterning events that result in a three-layered wall surrounding the AR, there is a period of anticlinal division that expands anther cell number and organ size [stage 4]. Subsequently, each SPC divides once periclinally to generate the ML and TAP and the final four somatic walled architecture of the pre-meiotic anther lobe is achieved [stages 5–7]. Prior to meiosis, anticlinal divisions occur to increase anther size, and the individual cell types acquire differentiated properties [stages 6–8], including dramatic enlargement of AR as they mature into Pollen Mother Cells (PMC) capable of meiosis [stage 8]. Comparison of anther lengths at the 8 stages plus meiotic entry in Arabidopsis (Smyth et al., 1990; Sanders et al., 1999), maize (Kelliher and Walbot, 2014; Zhang et al., 2014), and rice (Zhang et al., 2011) is summarized in the table; lengths marked with an (^*^) are inferred assuming linear growth in length in between known stages. **(C)** Longitudinal view of an anther lobe [stage 3] illustrates how AR column formation occurs simultaneously within both the tip and base of the lobe and that periclinal division of the PPC is stochastic. Parts of the illustrations in 1A,B are based on figures published in Zhang et al. (2014).

Successful anther development results in pollen dispersal. Steps required to achieve this are conveniently divided into three phases: organ patterning and initial cell differentiation, meiosis, and post-meiotic gametophyte development. Historically most studies have focused on meiosis and pollen biogenesis, with the assumption that a simple lineage model explained how the typical four somatic wall layers with central archesporial (AR) cells arose from a stamen primordium (Figure 1A). Starting with recovery and analysis of mutants defective in cell fate specification about 20 years ago (Sheridan et al., 1996; Canales et al., 2002; Zhao et al., 2002; reviewed by Ma, 2005), new theories and molecular insights into the first phase of anther development were proposed, disputed, and continue to be revised and elaborated.

## Early steps in anther ontogeny

The most detailed description of cellular numbers, shapes, and volumes during fate acquisition is available for maize utilizing 3-D reconstruction from confocal microscopy (Kelliher and Walbot, 2011) rather than transverse sectioning. Patterning to achieve the four somatic wall layers and central pre-meiotic cells typical of anther lobes is summarized in Figure 1B, employing pale coloration to indicate initial specification, with cell types darkening as differentiated features emerge. First, note that these stages, numbered one through eight, are processes, not discrete events. For example, starting from a primordium full of pluripotent cells, AR cells are the first to differentiate in anther lobes. In maize, discrete differentiation events from different precursors generate a column of ~10 AR cells over the course of 1 day. The first molecular marker of differentiating AR cells is MAC1 secretion; this ligand triggers pluripotent subepidermal cells to become bipotent Primary Parietal Cells (PPC) (Figure 1B, stage 2). The PPC then divide once periclinally to generate Endothecium (EN) and Secondary Parietal Layer (SPL) cells (Figure 1B, stage 3). In a given transverse section these steps occur successively, but viewing the anther longitudinally it is clear that AR differentiation in the anther base and tip occurs simultaneously with PPC periclinal divisions in the middle of the lobe (Figure 1C). Although it was long assumed that L1 presumptive epidermal cells and L2 internal cells have distinct fates locked in by their positions within an apical meristem, maize AR cells can differentiate from L1 cells during stage 2 low oxygen treatments: thus it appears that every maize anther primordium cell is pluripotent (Kelliher and Walbot, 2012). These observations lay to rest the lineage model where germinal and somatic cell fates diverge from a single “hypodermal cell” division event within each lobe.

Periclinal divisions generate new anther cell types and add cell layers to the anther wall, but most anther cell division is anticlinal (within layers). In maize a rapid elongation phase featuring exclusively anticlinal divisions prolongs stage 4 (Figure 1B), prior to SPL periclinal division into the Middle Layer (ML) and Tapetum (TAP) (Figure 1B, stages 5–7). This is followed by a second phase of rapid anther growth, prior to differentiation of post-mitotic AR cells into meiotically competent Pollen Mother Cells (PMC) (Figure 1B, stage 8). Although rice anthers are similar in size to maize during AR specification, both periods of rapid anticlinal cell division and expansion are absent (Figure 1B). As a result rice anthers starting meiosis are about one-third the length of maize; in rice, there is substantial anticlinal division during and just after meiosis. Arabidopsis anther primordia are considerably smaller than either grass—a combination of fewer, smaller cells—and like rice there is only modest pre-meiotic growth.

Given conserved internal anther anatomy, we expect similar regulatory processes among different flowering plants. Highlights of these common themes include: (1) many anther-specific mutants (Ma, 2005; Timofejeva et al., 2013); (2) a complex and dynamic transcriptome (Zhang et al., 2014); (3) communication between cell layers using secreted proteins (Wang et al., 2012); (4) presumptively locally produced hormones (Zhang et al., 2014), and (5) developmentally regulated small RNAs (Johnson et al., 2009). It is difficult to propose imposition of hormone or other gradients from materials delivered through the central vasculature (Figure 1A). Thus, unanswered questions include how the pace of anticlinal cell division is regulated autonomously within an anther lobe and what specific cues (activators and repressors) regulate periclinal divisions. It is striking that AR and later PMC development does not require normal somatic layers, as documented for the maize *mac1* mutant (Wang et al., 2012). Indeed, once specified, AR cells express a unique transcriptome, including precocious synthesis of transcripts for meiotic proteins, and robust production of transcripts for ribosomes and RNA binding proteins (Kelliher and Walbot, 2014). A major unanswered question is whether anther cell types express but then sequester mRNAs for use at later stages, a developmental mechanism widely employed in animal germlines (Zhang et al., 2014).

## Initial events resulting in AR column formation

A major breakthrough in plant reproductive genetics was identification of the Arabidopsis transcription factor *SPOROCYTELESS (SPL)* which was shown to be essential to AR cell differentiation and meiotic entry in both anthers and ovules (Yang et al., 1999). The late onset of AR-specific expression (~stage 4), however, suggested that *SPL* is only involved after initial fate acquisition, which at the time was thought to depend upon the inheritance of reproductive determinants via asymmetric periclinal division of a single founding hypodermal cell in each lobe. This lineage model ruled the field for decades, largely because early-acting mutants were challenging to identify and characterize, especially in Arabidopsis and rice. Then, in 2003 Chaubal et al. reported on a maize mutant, *male sterile converted anther1 (msca1)*, in which AR cells failed to form and were replaced by vascular bundles. When *MSCA1* was identified as a glutaredoxin (Albertson et al., 2009), and homologs were shown to affect AR fate acquisition in rice (Hong et al., 2012) and Arabidopsis (Xing and Zachgo, 2008), the first connection between protein redox status and plant reproductive cell fate was made.

**Figure 2:**
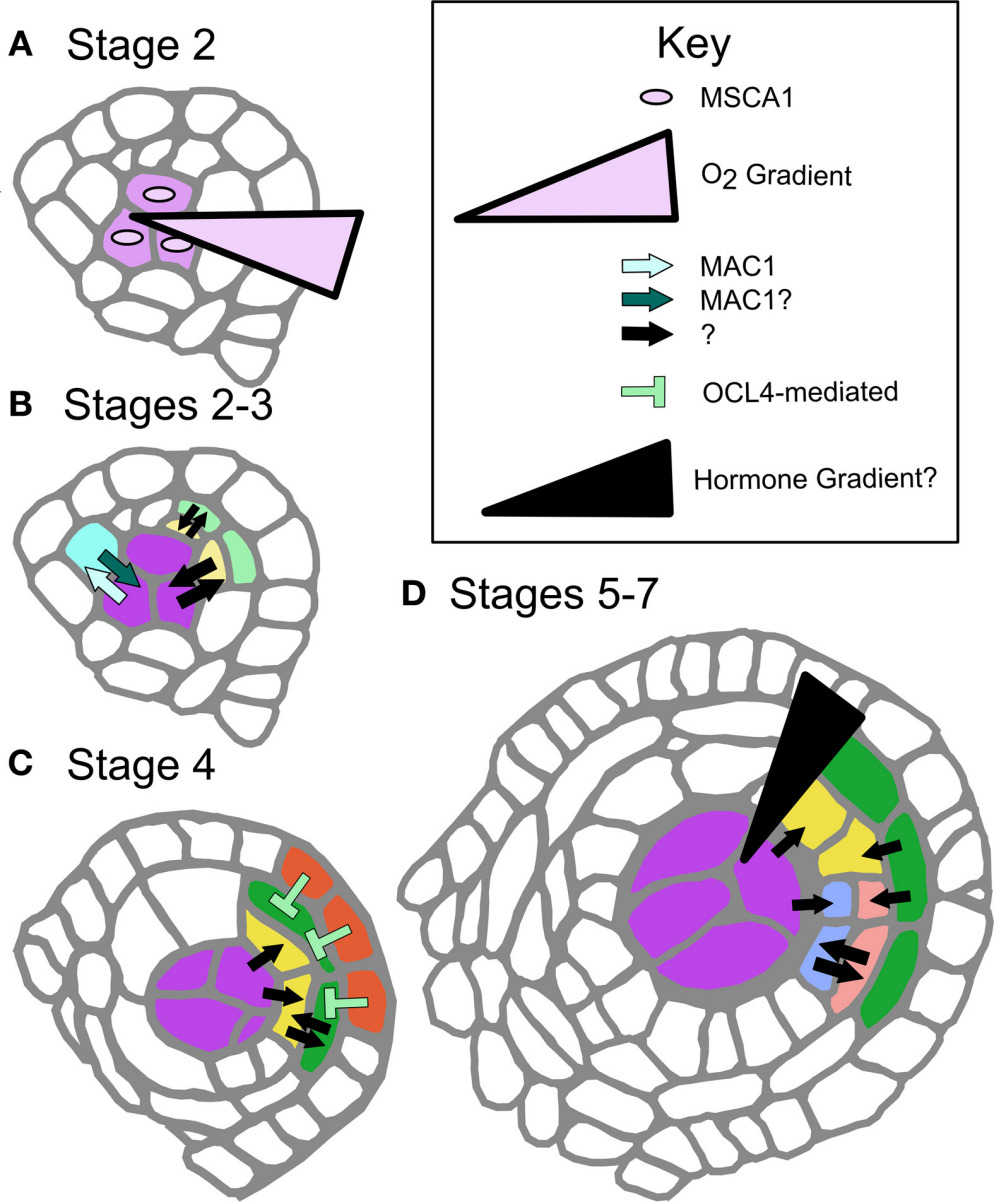
**Defined and proposed signaling networks in pre-meiotic maize development. (A)** Specification of AR cells is dependent on oxygen status interaction with MSCA1; hypoxic conditions stimulate AR cell differentiation. **(B)** As AR cells differentiate, they secrete the ligand MAC1, which is putatively perceived by LRR-RLK-type receptors on the L2-d cells, specifying these cells as soma and likely stimulating them to become PPC. MAC1 is also inferred to negatively regulate proliferation of AR cells until an entire column is formed in W23 (Wang et al., 2012). At these and later stages there are likely other cell-to-cell communication networks, indicated as black arrows with questions marks. These signals may be other ligand-receptor pairs, siRNAs, or other as yet undiscovered factors. **(C)** The differentiated EPI expresses transcription factor OCL4, which indirectly represses periclinal division in the neighboring EN, possibly by assisting with EN differentiation (Vernoud et al., 2009). **(D)** The trigger for periclinal division of the SPL, differentiation of the ML and TAP cell fates and maintenance of these fates is completely unexplored. A variety of cell-to-cell communication pathways might be in use (black arrows), or there may be a locally produced hormone gradient(s) along the Z-axis.

The application of confocal microscopy enabled a detailed morphometric analysis of AR column formation, which established that instead of just one founding germinal cell per lobe determined by inheritance, there were many initial AR cells specified from multiple progenitors. The lineage model was incorrect, and it was proposed that the internal position of AR cells within lobes determined their ultimate fate (Kelliher and Walbot, 2012). It was reasoned that such internal cells should be more hypoxic than their neighbors because of the high metabolic demand of rapid proliferation and the lack of air space in the tightly packed tissue (Figure 2A). To test the idea, hypoxia treatments were applied and rescued AR fate acquisition in *msca1*, dramatically increased AR cell counts in fertile anthers, and stimulated ectopic AR formation in both fertile and mutant anthers (Kelliher and Walbot, 2012). While redox manipulations were not attempted in other species, the mutant phenotypes of *Msca1* homologs in rice (*MIL1*) (Hong et al., 2012) and Arabidopsis (*ROXY1/ROXY2*) (Xing and Zachgo, 2008) indicate that glutaredoxin-based control of AR fate might be a conserved feature across flowering plants.

A major unanswered question is whether MSCA1 is expressed and active in the pluripotent L2-d cells surrounding the AR column, which experience mild hypoxia but will acquire a somatic fate. Glutaredoxins such as MSCA1 are known to bind and activate bZIP-type TGA transcription factors (Murmu et al., 2010); we hypothesize that among the MSCA1 targets are transcription factors that promote *Mac1* expression, the first molecular marker of AR fate acquisition. A secreted peptide, MAC1 signals pluripotent cells to differentiate as PPC (somatic) and divide periclinally forming the EN and SPL (Figure 2B). If MSCA1 is active in these presumptive PPCs, how does MAC1 signaling counteract or attenuate MSCA1 activity, overcoming mild hypoxia to enforce a somatic destiny? Likewise, does low oxygen tip the balance toward MSCA1 in the growing AR column, where newborn AR cells differentiate despite MAC1 secretion from existing AR cells (Figure 1C)? Ectopic AR cells arising from redox manipulation have column-forming abilities, even in *mac1*; therefore, column formation may be an emergent property of AR cells that is neither inhibited nor promoted by MAC1. Future work will continue to examine potential interactions between MSCA1 and MAC1 with respect to column formation.

Reproductive cell fate acquisition has put glutaredoxins at the forefront of the study of the genetic-environmental interface. These proteins appear to provide flexibility and responsiveness to hard-wired developmental programs and continue to be implicated in diverse plant developmental processes. In rice, the *mil1* glutaredoxin affects AR and SPL cell fate following AR differentiation (Hong et al., 2012) (stages 3–4), while the Arabidopsis glutaredoxin double mutant *roxy1/2* differentially affects ab- and ad-axial AR development (Xing and Zachgo, 2008), causing defects in stage 2 adaxial lobes and stage 7 abaxial lobes, implying a continued requirement for redox management during anther development and the involvement of multiple glutaredoxins in the differentiation of both germinal and somatic anther tissues. Mis-expression of MSCA1 in the shoot apical meristem gives opposite phyllotaxy in *Abph2* (Jackson et al., 2013), demonstrating the power of this protein to influence body plan elaboration. While genetic programming has a major role in early anther development—initial AR cell counts vary among inbred lines from ~10 in W23 to ~25 in A619—hypoxic treatments can double or triple initial AR counts without forcing a trade off in somatic cell populations. Therefore, the initial size of the AR population is controlled by both genetic factors and environmental conditions, and pollen production can be manipulated via genetic or extrinsic, redox-based treatments.

## Patterning the anther somatic niche

The somatic niche surrounding the developing AR cells is equally important for anther fertility. Contingent on MAC1-mediated signaling, L2-d cells are specified as somatic niche cells. The first morphological marker of somatic fate is PPC periclinal division that results in two somatic daughter cells (stage 3), that through subsequent periclinal divisions will differentiate as EN, ML, and TAP (Kelliher and Walbot, 2011; Timofejeva et al., 2013). Although EN, SPL, ML, and TAP cells are typically represented as differentiating immediately after a periclinal division, this is an over-simplification. Indeed, careful analysis of cell shape in maize indicates that these cells must expand in preferential dimensions before gradually achieving mature cell morphology (Kelliher and Walbot, 2011). Borrowing terminology from animal development, we consider somatic cells as passing through multiple steps, starting with cell fate specification, commitment, and finally differentiation. Periclinal division is the first marker of specification, and differentiation has occurred by the morphological end-point, but without deeper knowledge of cellular and molecular details, determining commitment timing is impossible. Does specification occur before a periclinal division, or after? When is commitment reached, and how are we to interpret the numerous mutants that persist in making periclinal divisions, i.e., *ocl4*, *ms23*, and *ms32* in maize (Chaubal et al., 2000; Vernoud et al., 2009; Moon et al., 2013, Figure 2C), and *tdf1* in Arabidopsis (Zhu et al., 2008)?

A second issue is within-layer stochasticity in periclinal division initiation. AR cells initiate meiosis synchronously, yet the periclinal divisions resulting in the EN/SPL and later the ML/TAP are both asynchronous and exhibit no discernible spatial pattern (Kelliher and Walbot, 2011; Zhang and Li, 2014). Each cell appears to divide periclinally after a variable number of anticlinal divisions. Could signals directing periclinal divisions be perceived during a restricted portion of the cell cycle, resulting in a large population of cells deaf to the signal when first available? Asynchrony of anticlinal somatic division would then result in asynchrony of periclinal divisions.

The complexity and lack of information on signaling within the soma makes investigations into the regulation of somatic cell patterning and specification in the anther ripe for pursuit. Despite the relative dearth of information, there are some hints that allow predictions (Figure 2). First, the secreted peptide and receptor model may be a common theme. Mutants in Arabidopsis and rice homologs *AtTPD1* and *OsTDL1A* have excess AR cells but develop a normal EN and SPL (stage 5, Figure 1B) (Feng and Dickinson, 2010). Clues from these mutants suggest the MAC1 peptide family binds LRR-RLK receptors (*AtEXS/EMS1* and *OsMSP1*, respectively). As LRR-RLKs are known to dimerize, hetero-dimerization utilizing different isoforms or family members may contribute to differential signal interpretation in peripheral L2-d and presumptive AR cells during column formation, or between other cell types at later stages (Wang et al., 2012). Elucidating the suite of LRR-RLKs could uncover a distinct “combinatorial code” of dimeric receptors on different cell types. This idea is consistent with the presence of many putatively secreted peptides (Wang et al., 2012) and the expression of numerous LRR-RLKs in anthers (Kelliher and Walbot, 2014; Zhang et al., 2014). The anther is one of many organs where peptide signaling has emerged as a major developmental paradigm. In particular, tissues that lack direct access to vasculature, such as stem cells in the shoot apical meristem and meristemoid cells of the stomatal lineage (Li and Torii, 2012), often employ this mode of cell-cell communication.

The role of hormones or other chemical gradients within anther lobes is another area yet to be explored on a cell type-specific level. In a commonly cited example, periclinal division in Arabidopsis roots involves an auxin gradient delivered by polarized auxin flow (Cruz-Ramirez et al., 2012). Given the anatomy of the anther, it is unlikely that a similar flow exists because cell types are not a uniform distance from the vasculature. Local hormone production and perception would more likely be a feature of anther development (Figure 2D). Transcriptome analysis indicates that both gibberellin and brassinolide associated genes are differentially regulated in stage 7 pre-meiotic anther development; these would make strong initial candidates for further exploration as regulators of anther cell fates (Zhang et al., 2014).

Despite identification of many maize male-sterile mutants disrupting pre-meiotic somatic patterning (Table 1), this phase of early anther development is underrepresented in the rice and Arabidopsis literature. The small size of rice and Arabidopsis anthers at these stages (20–70 and 100–350 μm, respectively, compared to 120–700 μm for maize) along with the rapidity of development (in maize, events span nearly 3 days, roughly twice as long as Arabidopsis) makes isolation and precise analysis of discrete stages difficult (Figure 1B). Many mutants that fail to complete meiosis have been categorized as meiotic mutants, even when the defect is earlier and the primary defect is in the soma. There is no consensus concerning the specific roles of each somatic cell type. The tapetum has been characterized as a highly transcriptionally active, secretory cell type required for exine formation. But the ML can be present as either a single layer (as in rice, maize, and Arabidopsis), or several layers, but is always consistent within a species (Esau, 1965; D'Arcy and Keating, 1996). Middle-layer like tissue and dartboard lobe architecture are relatively ancient, dating to the gymnosperm microsporangia (Esau, 1965; D'Arcy and Keating, 1996), but as of yet no specific function has been proposed for the ML. Secondary wall thickening of the EN is involved in anthesis at the end of anther development, but no attention has been given to earlier roles. Germ cell establishment and the subset of tapetal mutants that result in meiotic arrest have received the vast majority of attention.

**Table 1:** **A comprehensive list of anther mutants sequentially organized from organ specification through meiosis**.

**Stage affected**	***A. thaliana***	**Rice**	**Maize**	**Annotation**	**Phenotype**	**doi**
1	*agamous*	*mads3, mads58*	*Zmm2, Mads2*	MADS-box transcription factor	stamens converted to petals (Arabidopsis) or lodicules (grasses)	10.1105/tpc.3.8.749
1			*ems71924, ems72032*	*not cloned*	stamen adaxialization	10.1534/g3.112.004465
1			ems71990, ms-si^*^355	*not cloned*	absence of anthers in some florets	10.1534/g3.112.004465
1	*rdr6*	*rol (SHOOTLESS2)*	*rdr6*	RNA-directed RNA polymerase	stamen abaxialization (defect in tasi-ARF biosynthesis)	10.1105/tpc.110.075291
2	*bam1 bam2 double mutant*			LRR receptor-like kinases	all internal lobe cells become AR; no somatic cells	10.1105/tpc.105.036871
2		*mil1*	*msca1*	glutaredoxin	AR fail to differentiate (*Os*) or differentiate as vasculature (*Zm*)	10.1007/s00425-002-0929-8
2 (ad), 8 (ab)	*roxy1 roxy2 double mutant*			glutaredoxin (thioreductase)	adaxial lobes: AR specification failure; abaxial: PMC degrade	10.1111/j.1365-313X.2007.03375.x
3	*nzz = spl*	no homology in grasses		MADS-box transcription factor	AR differentiation failure; somatic cell layer defects	10.1104/pp.109.145896
3 (*Zm*/*Os*), *5* (*At*)	*tpd1*	*tdl1a = mil2*	*mac1*	small secreted protein ligand	somatic cell specification failure; overproliferation of AR	10.1105/tpc.016618
3	*exs = ems1*	*msp1*		LRR receptor-like kinase	somatic cell specification failure; overproliferation of AR	10.1016/S0960-9822(02)01151-x
4			*ocl4*	HD-ZIP IV transcription factor	additional periclinal divisions in subepidermal cell layer	10.1111/j.1365-313X.2009.03916.x
4			*ems63089, tcl1, mtm00-06*	*not cloned*	undifferentiated somatic cell layers	10.1534/g3.112.004465
5		*tip2*		bHLH transcription factor	all three anther wall layers fail to differentiate properly	10.1105/tpc.114.123745
5	*er erl1 erl2 triple mutant*			LRR receptor-like kinases	missing anthers and somatic cell differentiation defects	10.1093/mp/ssn029
6	*tdf1*			R2R3 Myb transcription factor	early vacuolization in epidermis and endothecium, tapetal failure	10.1111/j.1365-313X.2008.03500.x
6	*serk1 serk2 double mutant*			LRR receptor-like kinases	SPL periclinal division failure	10.1105/tpc.105.036731
7			ms23, ms^*^6015	*not cloned*	additional periclinal divisions in the tapetal layer	10.1534/g3.112.004465
7			*ems72063*	*not cloned*	undifferentiated soma; excess periclinal divisions in tapetum	10.1534/g3.112.004465
7			*ems72091*	*not cloned*	additional periclinal divisions in the middle layer	10.1534/g3.112.004465
7	*mpk3 and mpk6*			MAP kinases	somatic cell specification failure; overproliferation of AR	10.1093/mp/ssn029
8		*dtm1*		ER membrane protein	tapetal differentiation failure	10.1111/j.1365-313X.2011.04864.x
8	*dyt1*			bHLH transcription factor	tapetal differentiation failure	10.1111/j.1365-313X.2012.05104.x
8	*myb33 myb65 double mutant*			GAMYB-like transcription factor	tapetal differentiation failure	10.1105/tpc.104.027920
8			*ms9, ms11, ms13, ms14*	*not cloned*	tapetal differentiation failure	10.1534/g3.112.004465
8			*ms32*	bHLH transcription factor	excess periclinal divisions in tapetum after normal wall is built	10.1111/tpj.12318
8			*csmd1*	*not cloned*	excess pre-meiotic callose and slow dissolution of the tetrad	10.1007/s00497-011-0167-y
8			*ms8*	beta-1,3-galactosyl transferase	cell growth defects in epidermis and tapetum, meiotic arrest	10.1007/s00497-013-0230-y
8		*gamyb-4*		GAMYB transcription factor	tapetal differentiation failure; meiotic arrest	10.1111/j.1744-7909.2010.00959.x
meiosis		*udt1*		bHLH transcription factor	tapetal differentiation failure; meiotic arrest	10.1105/tpc.105.034090
meiosis		*mel1*		Argonaute	tapetal differentiation failure; meiotic arrest	10.1105/tpc.107.053199

The left hand column indicates the first developmental stage at which a mutant phenotype is observed, using the staging rubric outlined in Figure 1. In cases where the onset of the phenotype differs among species or tissues, abbreviations are used (“Zm” = maize, “Os” = rice, “At” = Arabidopsis, “ab” = abaxial anther lobes, and “ad” = adaxial anther lobes). If a given mutant is phenocopied by homologs from the other two species, the gene names are given in the corresponding species' column. An exception to this rule was made for the first maize entry, “Zmm2, Mads2,” because while mutants in these genes have not been found, the genes are clearly agamous orthologs by sequence comparison and expression pattern in the third floral whorl, which is characteristic of C class genes. The next two columns contain the phenotypic description of the mutant and protein annotation if a causative gene has been cloned. Uncloned mutants are indicated as “not cloned,” and these are clustered in a single row in cases where they roughly phenocopy each other (for example, maize ems71924 and ems72032 have nearly identical anther polarity phenotypes, and may be allelic). In the final column, a doi is provided for the founding mutant of each class. The high number of blank spaces in the species' columns reflects the challenge of comparisons between model species. The bHLH, MYB, and LRR-RLK genes are all found in large families making identification of orthologs between species problematic. Furthermore, mutations in a single gene that cause a clear phenotype in one plant species may not be available in others because of functional gene redundancy from lineage specific gene duplication. And there is already evidence that orthologs can regulate different steps reflecting evolutionary diversification of developmental pathways. For these reasons we do not anticipate a high degree of correspondence between Arabidopsis, rice, and maize.

## Role of small RNAs in anther patterning

Developing anthers depend on small RNAs for gene regulation, cell-to-cell communication, and epigenetic reprogramming. For example, in rice, anther adaxial-abaxial polarity is regulated by trans-acting siRNAs (tasiRNAs), which are secondary siRNAs derived from *TAS* transcripts (Toriba et al., 2010). These transcripts are first processed by miRNA-guided cleavage and then converted to double-stranded RNAs by RNA-dependent RNA polymerase6 (RDR6), followed by Dicer4 (DCL4) cleavage yielding 21-nt tasiRNAs (Allen et al., 2005). Mutants that disable components of the tasiRNA biogenesis pathway display severe defects in floral morphology and fertility. Rice *dcl4* exhibits a disruption in lemma abaxial-adaxial polarity and abnormal anther development (Liu et al., 2007). In the *rod-like lemma (rol/rdr6)* mutants, stamen, and lemma development are severely compromised (Toriba et al., 2010). A single nucleotide polymorphism in the same gene results in reduced fertility at high temperature (Song et al., 2012a). The phenotypes of these mutants are largely caused by loss of tasiRNAs, resulting in upregulation of target *Auxin Response Factor* genes required for abaxial identity (Pekker et al., 2005).

Recently two size classes of phased siRNAs (phasiRNAs) were found to be preferentially expressed in grass inflorescences, particularly in anthers (Johnson et al., 2009; Song et al., 2012b). Both phasiRNA types are derived from low copy intergenic regions, and each requires a specific miRNA trigger (miR2118 for the 21-nt class and miR2275 for 24-nt class) to initiate cleavage and RDR6-dependent second-strand synthesis. The resulting double-stranded RNAs are then cleaved by DCL4 to produce 21-nt phasiRNAs and by DCL5 for 24-nt phasiRNAs (Arikit et al., 2013). Despite similarities to tasiRNA biogenesis, the functions of these 21- and 24-nt phasiRNAs and their targets are largely unknown. The phasiRNAs lack complementarity to genes or transposons, suggesting novel roles rather than post-transcriptional gene silencing or RNA-directed DNA methylation.

Post-meiotically, small RNAs do provide germinal cell genome surveillance in microspores. Triggered by miRNAs and dependent on RDR6 and DCL4 (Creasey et al., 2014), the 21-nt epigenetically-activated siRNAs (easiRNAs) are derived from transposon transcripts exclusively expressed in the vegetative nucleus of Arabidopsis pollen grains; easiRNAs then move to sperm cells to direct transcriptional gene silencing (Slotkin et al., 2009). As a result, sperm cell chromatin is highly condensed and enriched in the repressive epigenetic modifications while chromatin in the vegetative cell nucleus is largely decondensed (Slotkin et al., 2009).

Despite sharing biogenesis factors including DCL4 and RDR6, 21-nt phasiRNAs are different from 21-nt easiRNAs in three ways. First, although 21-nt phasiRNAs have been found in dicots, they are mostly derived from a single *NB-LRR* defense gene family (Zhai et al., 2011); in contrast hundreds of grass loci encode phasiRNAs. Second, phasiRNAs are produced prior to meiosis or gametogenesis (Komiya et al., 2014) while easiRNAs are found specifically in gametophytes. Third, easiRNAs are derived from repetitive regions, while phasiRNAs are produced from unique or low copy sequences. These distinctions between phasiRNAs and easiRNAs suggest their functional divergence and imply a sporophytic role for phasiRNAs in anther development.

ARGONAUTE proteins have diverged rapidly in plants, with 10 members in Arabidopsis (Chen, 2009), 19 in rice (Kapoor et al., 2008), and 17 in maize (Zhai et al., 2014); many are preferentially expressed in germinal cells (Zhai et al., 2014). The continued diversification of ARGONAUTEs suggests that many new functions for small RNAs during plant reproduction await discovery. MEL1, a rice homolog of Arabidopsis AGO5, mainly localizes to the cytoplasm of pre-meiotic sporocytes. *mel1* loss-of-function mutants exhibit abnormal tapetal formation and contain aberrant PMC that arrest in early meiosis (Nonomura et al., 2007). Recently MEL1 was demonstrated to bind 21-nt phasiRNAs preferentially (Komiya et al., 2014). Histone methylation patterns are altered in *mel1* mutant meiocytes (Nonomura et al., 2007), hinting at a role for MEL1 and its associated 21-nt phasiRNAs in chromatin modification. Because the tapetal somatic defect precedes the documented meiocyte phenotypes in *mel1* anthers, it is not yet clear if MEL1/21-nt phasiRNAs act directly or indirectly to disrupt meiosis.

Maize *AGO104* is a homolog of Arabidopsis *AGO9* and its transcripts accumulate specifically during pre-meiosis just following germinal differentiation in anthers (Kelliher and Walbot, 2014) and during meiosis (Singh et al., 2011). Maize *ago104* mutants show defects in meiotic chromatin condensation and subsequent failure to properly segregate chromosomes (Singh et al., 2011). Small RNAs interacting with AGO104 are yet to be profiled, but the heterochromatic decondensation in maize *ago104* mutants suggests a role in germinal cell epigenetic reprogramming. With rapid progress in deep sequencing of small RNAs and RNA-IP-seq, we are likely to acquire a better understanding of the diverse ways small RNAs contribute to anther development and plant reproduction.

## Author contributions

Each author wrote one section of the manuscript, edited all sections, and approved final submission.

### Conflict of interest statement

Timothy Kelliher and Virginia Walbot are inventors on a patent application (61/598.544) filed by Stanford University entitled “Method for Modulating the Number of Archesporial Cells in a Developing Anther.” The authors declare that the research was conducted in the absence of any commercial or financial relationships that could be construed as a potential conflict of interest.
